# Transkingdom Analysis of the Female Reproductive Tract Reveals Bacteriophages form Communities

**DOI:** 10.3390/v14020430

**Published:** 2022-02-19

**Authors:** Ferralita S. Madere, Michael Sohn, Angelina K. Winbush, Breóna Barr, Alex Grier, Cal Palumbo, James Java, Tracy Meiring, Anna-Lise Williamson, Linda-Gail Bekker, David H. Adler, Cynthia L. Monaco

**Affiliations:** 1 Department of Microbiology and Immunology, University of Rochester Medical Center, Rochester, NY 14642, USA; ferralita_madere@urmc.rochester.edu; 2 Department of Biostatistics and Computational Biology, University of Rochester Medical Center, Rochester, NY 14642, USA; michael_sohn@urmc.rochester.edu; 3 Division of Internal Medicine, University of Rochester School of Medicine & Dentistry, Rochester, NY 14642, USA; angelina_winbush@urmc.rochester.edu; 4 Department of Rural Family Medicine, West Virginia University, Morgantown, WV 25425, USA; breona.barr@hsc.wvu.edu; 5 UR Genomics Research Center, University of Rochester Medical Center, Rochester, NY 14642, USA; akg2685@rit.edu (A.G.); cal_palumbo@urmc.rochester.edu (C.P.); james_java@urmc.rochester.edu (J.J.); 6 Institute of Infectious Diseases & Molecular Medicine and Division of Medical Virology, Faculty of Health Sciences, University of Cape Town, Anzio Road, Observatory, Cape Town 7925, South Africa; tracy.meiring@gmail.com (T.M.); anna-lise.williamson@uct.ac.za (A.-L.W.); 7 South African Medical Research Council Gynaecological Cancer Research Centre, Faculty of Health Sciences, University of Cape Town, Cape Town 7505, South Africa; 8 Desmond Tutu HIV Centre, Institute of Infectious Diseases & Molecular Medicine, Faculty of Health Sciences, University of Cape Town, Anzio Road, Observatory, Cape Town 7925, South Africa; linda-gail.bekker@hiv-research.org.za; 9 Department of Emergency Medicine, University of Rochester Medical Center, Rochester, NY 14642, USA; david_adler@urmc.rochester.edu; 10 Department of Internal Medicine, Division of Infectious Diseases, University of Rochester Medical Center, Rochester, NY 14642, USA

**Keywords:** virome, microbiome, bacterial vaginosis, bacteriophage, transkingdom associations, female reproductive tract, HIV

## Abstract

The female reproductive tract (FRT) microbiome plays a vital role in maintaining vaginal health. Viruses are key regulators of other microbial ecosystems, but little is known about how the FRT viruses (virome), particularly bacteriophages that comprise the phageome, impact FRT health and dysbiosis. We hypothesize that bacterial vaginosis (BV) is associated with altered FRT phageome diversity, transkingdom interplay, and bacteriophage discriminate taxa. Here, we conducted a retrospective, longitudinal analysis of vaginal swabs collected from 54 BV-positive and 46 BV-negative South African women. Bacteriome analysis revealed samples clustered into five distinct bacterial community groups (CGs), and further, bacterial alpha diversity was significantly associated with BV. Virome analysis on a subset of baseline samples showed FRT bacteriophages clustering into novel viral state types (VSTs), a viral community clustering system based on virome composition and abundance. Distinct BV bacteriophage signatures included increased alpha diversity along with discriminant *Bacillus*, *Burkholderia,* and *Escherichia* bacteriophages. Bacteriophage-bacteria transkingdom associations were also identified between *Bacillus* and *Burkholderia* viruses and BV-associated bacteria, providing key insights for future studies elucidating the transkingdom interactions driving BV-associated microbiome perturbations. In this cohort, bacteriophage-bacterial associations suggest complex interactions, which may play a role in the establishment and maintenance of BV.

## 1. Introduction

The female reproductive tract (FRT) houses a compositionally dynamic environment where the host participates in an intricate interplay with a microbiome composed of bacteria and archaea (bacteriome), fungi (fungome), viruses (virome), and occasional protozoal parasites [1,2]. The FRT microbiome plays an important protective role in maintaining vaginal health and preventing urogenital diseases such as bacterial vaginosis (BV), yeast infections, pre-term birth, and sexually transmitted infections (STIs), including human immunodeficiency virus (HIV) [3,4,5,6]. Prior studies of the FRT microbiome have primarily focused on determining bacterial composition and function. At least five different bacterial community groupings have been described within the FRT, distinguishable by the dominance of *Lactobacillus* species or the presence of more diverse anaerobes [7,8]. The prevalence of these communities varies by race and ethnic group, with the majority of Caucasian women hosting *Lactobacillus*-dominant FRT microbiomes, whereas African women tend to be asymptomatically colonized by higher diversity FRT microbiota [8,9]. *Lactobacillus*-dominant FRT bacteriomes, especially *L. crispatus*, protect against vaginal disease through several mechanisms, including by competitive exclusion of pathogenic bacteria for space and nutrients, promoting an acidic vaginal environment via production of lactic acid, and maintaining a low inflammatory state [10,11,12]. BV, the most common cause of vaginal discharge in reproductive age women, is a symptomatic clinical condition characterized by a shift in the FRT microbiota away from a low inflammatory, *Lactobacillus*-dominant bacteriome to a more diverse community including facultative anaerobes. BV is associated with an increased risk of STI acquisition and pre-term birth [13,14]. Specific BV-associated bacteria include *Gardnerella vaginalis*, *Prevotella*, *Fusobacterium*, *Atopobium vaginae*, *Megasphaera*, and *Sneathia,* among others [15]. FRT bacteriome shifts can occur rapidly and may be related to shifts in bacteriophage populations [16,17].

Viruses rival bacterial numbers in the microbiome and are more diverse [18]. However, studies of the virome have been limited, in part due to the lack of a common viral genetic element analogous to the bacterial 16S rRNA gene, as well as the high genetic diversity between viral species [18,19]. The FRT virome is home to eukaryotic viruses and bacteriophages [18]. Compared to the FRT bacteriome, little is known about the viral communities of the FRT or how their interactions with bacteria contribute to disease states such as BV. The few prior studies that have examined the FRT virome have mainly concentrated on the deoxyribonucleic acid (DNA) eukaryotic virome, finding *Papillomaviridae, Polyomaviridae, Herpesviridae, Poxviridae, Adenoviridae*, and *Anelloviridae* present [20,21]. However, bacteriophages that make up the phageome are the largest and most abundant viral group and can modulate bacterial composition and abundance, suggesting that they may play an important role in regulating bacterial composition of the FRT microbiome [19,22]. Bacteriophages may be lytic, hijacking bacterial host replication machinery in order to replicate and then lysing the host to release virions, or lysogenic, whereby bacteriophage DNA is integrated into the host genome as a prophage and replicates in tandem with bacterial genome replication [23]. This lysogenic lifestyle can result in generalized transduction of bacterial genes between bacterial hosts that can confer increased fitness through methods such as toxin production, carbohydrate metabolism, or antibiotic resistance [24]. Upon environmental stress, lysogenic bacteria can become lytic and, therefore, may serve to regulate bacterial populations in unfavorable host conditions.

Data on bacteriophage populations in the FRT are limited, with studies in general hampered by low sequencing depth that prevents a thorough characterization of the FRT phageome [1]. One of the earliest studies revealed numerous *Caudovirales* order bacteriophages in the FRT; however, no relationship was found between FRT *Caudovirales* bacteriophages and bacterial populations [4]. Another group examining a cohort of BV-positive and BV-negative Danish women found no significant difference in viral (including bacteriophage) nor bacterial alpha diversity between BV-positive and BV-negative women [25]. A recent investigation of FRT bacteriophages in South African adolescents showed prevalence and persistence of prophages, but the sample size was small, with only 13 participants [26]. A more in-depth examination of FRT bacteriophage populations is needed to identify bacterial-bacteriophage perturbations in health and disease.

Herein we investigated FRT virome composition and transkingdom bacterial-bacteriophage associations within the FRT. We assessed the vaginal microbiome of 100 young, sexually active BV-positive and BV-negative South African women to identify discriminate viral and bacterial signatures in health and FRT disease. We show for the first time that FRT DNA bacteriophage populations cluster into community groupings that correspond to bacterial community groupings and that specific bacteriophages correlate with bacteria associated with and protective against BV. These findings improve our understanding of the transkingdom associations in the FRT microbiome and the impact that these could have on the induction and pathogenesis of disease.

## 2. Materials and Methods

### 2.1. Study Cohort

De-identified vaginal swabs from the University of Cape Town HPV-HIV study [27] were retrospectively used for bacteriome and virome analysis. This cohort was comprised of 50 HIV-positive and 50 HIV-negative young, sexually active women between the ages of 16 and 21 recruited from the youth community center and clinic in two urban communities (Masiphumelele and Mthatha townships) in Cape Town, South Africa, between October 2012 and October 2014. Informed consent was obtained from all participants above 18 years of age, and parental consent was obtained for participants of age 17 or younger. This study was approved by the Institutional Review Board at the University of Rochester Medical Center and the Human Research Ethics committee at the University of Cape Town. Vaginal swabs were self-collected approximately every 6 months by subjects using Dacron swabs high within the vagina, placed in Digene transport media, and frozen at −80 °C until use. Pap smears were taken at baseline and at least one other visit, and tests for human papilloma virus (HPV), *Trichomonas*, and BV were performed [27]. HIV status was confirmed upon enrollment, and CD4+ T cell count for those who were HIV-positive was determined. Exclusion criteria included prior HPV vaccination or cervical surgery. Sexual history, recent contraceptive methods, and current HIV treatment interventions were also queried at study entry.

### 2.2. Bacterial 16S rRNA Gene Amplicon Sequencing

Total nucleic acid was extracted from 253 resuspended vaginal swab samples using the MagNA Pure Compact Nucleic Acid Isolation Kit (Roche) [27]. The 16S rRNA gene amplicon sequencing was performed with primers specific to the V3-V4 region [28], followed by amplicon pooling, bead-based normalization, and sequencing on the Illumina MiSeq platform at 312 bp paired-end reads (University of Rochester Genomics Research Center, UR GRC). Water processed similarly to samples and pre-defined bacterial mixtures (Zymo) were run as negative and positive controls, respectively. Eleven samples failed 16S rRNA gene amplification.

### 2.3. Bacterial 16S rRNA Gene Amplicon Analysis

Raw data from the Illumina MiSeq were first converted into FASTQ format 2 × 312 paired-end sequence files using the bcl2fastq program (v1.8.4) provided by Illumina. Format conversion was performed without de-multiplexing, and the EAMMS algorithm was disabled. All other settings were default. Reads were multiplexed using a configuration described previously [28]. The extract_barcodes.py script from QIIME (v1.9.1) [29] was used to split read and barcode sequences into separate files suitable for import into QIIME 2 (v2018.11) [30], which was used to perform all subsequent read processing and characterization of sample composition. Reads were demultiplexed requiring exact barcode matches, and 16S primers were removed, allowing 20% mismatches and requiring at least 18 bases. Cleaning, joining, and denoizing were performed using DADA2 [31]: reads were truncated (forward reads to 260 bps and reverse reads to 240 bps), error profiles were learned with a sample of one million reads per run, and a maximum expected error of two was allowed. Taxonomic classification was performed with custom naïve Bayesian classifiers trained on target-region-specific subsets of the August 2013 release of Greengenes [32]. Sequence variants that could not be classified to at least the phylum level were discarded. Sequencing variants observed fewer than ten times total or in only one sample were discarded. Vaginal samples with fewer than 10,000 reads and/or features present in less than 20 samples were discarded. Four samples and all negative controls did not achieve sufficient sequence variants for downstream analysis, leaving 238 samples that were included in the final analysis. Phylogenetic trees were constructed using MAFFT [33] for sequence alignment and FastTree [34] for tree construction. For the purposes of diversity analyses, samples were rarefied to a depth of 10,000 reads. Faith’s PD and the Shannon index were used to measure alpha diversity, and weighted UniFrac [35] was used to measure beta diversity. Assignment of FRT bacterial community state types (CSTs) was performed using VALENCIA [36]. Taxonomy was assigned to the 16S sequences using a Greengenes 99% OTUs trained classifier [32]. The QIIME2 ASV taxonomy and ASV read count table were converted to a VALENCIA suitable format using a conversion script provided by the VALENCIA authors. VALENCIA was then run using the author provided “CST_centroids_012920” as the reference centroids.

### 2.4. Lactobacilli DNA Extraction and qPCR Analysis

Quantitative PCR (qPCR) primers targeting the 16S rRNA genes of *L. iners*, *L. crispatus*, *L. gasseri*, and *L. jensenii* were previously designed in [37]. DNA was extracted from vaginal swabs as described above for 16S rRNA sequencing [38]. To serve as positive controls, generate standard curves, and measure the detection limit of species-specific 16S rRNA gene qPCR assays, *Lactobacillus iners* (ATCC 55195), *Lactobacillus crispatus* (ATCC 33820), *Lactobacillus gasseri* (ATCC 33323), and *Lactobacillus jensenii* (ATCC25258) genomic DNA was extracted using isopropanol precipitation. The 10-fold serial dilutions (10^0^ to 10^8^ copies) were used to generate standard curves of extracted genomic DNA. The range of slopes for the qPCR assays was from −3.7 to −4.7, and r^2^ values were all >0.99. SYBR green-based qPCR assays were performed on a CFX96 Touch Real-Time PCR Detection System (Bio-Rad, Hercules, CA, USA). The reaction mixture (20 µL) contained 10 µL iQ SYBR Green Supermix (Bio-Rad), 2 µL each of forward and reverse primers to a final primer concentration of 100 nM each except for the assay for *L. iners,* which had used 4 µL each to a concentration of 200 nM., and 10 µL template DNA (10 ng). Temperature cycling for all assays was polymerase activation at 95 °C for 3 min, followed by 40 cycles of amplification with denaturation at 95 °C for 15 s, followed by annealing/ extension at 55 to 58 °C for 1 min (*L. gasseri*, *L. iners* and *L. jensenii* at 55 °C and *L. crispatus* at 58 °C). Fluorescence was measured at the final step of each cycle. For each qPCR assay, vaginal swabs and extracted lactobacilli genomic DNA standards were run in triplicate, and the average values were used to calculate 16S rRNA gene copy number per 10 ng total vaginal sample DNA. Negative (no DNA water) controls were run with every assay to check for contamination.

### 2.5. Virus-Like Particle Preparation, Library Construction, and Sequencing

Virus-like particle (VLP) preparation was adapted from methods previously described [39]. Briefly, vaginal specimens were resuspended in 200 μL of Digene transport media, and an equal volume of SM buffer was added (50 mM Tris-HCl, 8 mM magnesium sulfate, 100 mM sodium chloride, and 0.01% gelatin, pH 7.5, Fisher) and mixed by vortexing for 5 min. Specimens were centrifuged at 2000× *g* and filtered using a 0.45 μM filter to remove intact cells and bacteria. Samples underwent lysozyme treatment (1 μg/mL at 37 °C for 30 min) (Sigma-Aldrich) to degrade the remaining host cell and bacterial membranes. DNase digestion (Turbo DNase Buffer, Turbo DNase, Baseline Zero) (Ambion) was performed to remove contaminating bacterial and host DNA followed by heat inactivation of the DNase at 75 °C for 15 min. Enriched VLPs were lysed with 10% SDS and 20 mg/mL proteinase K (Ambion) at 56 °C for 20 min, followed by treatment with CTAB (10% Cetyltrimethylammonium bromide, 0.5 M NaCl, nuclease-free water, filtered through 0.22 μM filter) at 65 °C for 20 min. Phenol: chloroform: isoamyl alcohol (Invitrogen, pH 8.0) nucleic acid extraction was performed, the resulting aqueous fraction then washed with an equal volume of chloroform and concentrated through isopropanol precipitation. Extracted nucleic acid was aliquoted and stored at −80 °C until use. NEBNext Ultra II FS DNA Library Prep Kit (New England Biolabs) was used for library construction with NEBNext Multiplex Oligos for Illumina Dual Index Primers (New England Biolabs). Following equimolar pooling, DNA libraries were sequenced on the Illumina NovaSeq platform (UR GRC), generating an average of over 29 million 150 bp paired-end reads per sample.

### 2.6. Virome Analysis Pipeline

The VirusSeeker pipeline [40] was deployed on a Linux cluster on raw sequence data. Briefly, raw sequences from samples went through sample pre-processing steps that included adapter removal, stitching of reads, quality filtering, and CD-HIT was used to minimize sequence redundancy and define unique sequences (98% identity over 98% of the sequence length). Sequencing reads underwent human genome filtering, then unmapped reads were sequentially queried against a customized viral database comprised of all viral sequences in NCBI using BLASTn (e-value cutoff 1 × 10^−10^), followed by BLASTx (e-value cutoff: 1 × 10^−3^). False-positive viral sequences were identified by successively querying the candidate viral reads against the NCBI NT database using MegaBLAST (e-value cutoff: 1 × 10^−10^), BLASTn (e-value cutoff: 1 × 10^−10^), and the NCBI NR database using BLASTx (e-value cutoff: 1 × 10^−3^). All sequences that aligned to viruses were further classified into viral genera and species based on the NCBI taxonomic identity of the top hit.

### 2.7. Viral Assembly and Lytic/Lysogenic Gene Determination

Reads were preprocessed using KneadData for quality and contaminating human sequence removal [41]. Viral contigs were assembled from the quality-controlled reads using Megahit [42]. Prodigal was used to identify protein-coding genes [43]. To determine the presence of viral proteins, HMMER [44] was used to search for sequence homologs among the prokaryotic virus orthologous groups (pVOGs) database [45], which represents a comprehensive set of orthologous gene families shared across multiple complete genomes of viruses that infect bacterial or archaeal hosts. Contigs with identified proteins consistent with lysogenic bacteriophage (integrase, transposase, repressor, and recombinase proteins) were counted as lysogenic; contigs with identified proteins matching to lytic lifecycle (lysin, antirepressor, and holin proteins) with no identified lysogenic genes present were counted as lytic.

### 2.8. Statistical Analysis

Descriptive statistics were used to summarize the characteristics of the study population. Mean was used for continuous variables, and frequency or proportion was used for categorical variables. Alpha diversity was measured by Shannon’s diversity index and analyzed using a linear mixed-effects model for bacteria data and a linear regression model for bacteriophage data [46]. Beta diversity was measured by the weighted UniFrac distance for bacteria data and by the Bray–Curtis distance for bacteriophage data [47,48]. Permutational multivariate analysis of variance (PERMANOVA) was used to quantify dissimilarity in beta diversity [49]. Hierarchical clustering of samples into distinct bacterial and bacteriophage community profiles, community groups, and viral state types, respectively, was performed using partitioning around medoids per Ward’s linkage method, with the number of clusters estimated by maximum average silhouette width [50]. For differential abundance analysis, the relative abundance was first arcsine-transformed, and then univariate analysis was performed using mixed-effects models for bacteria data and linear regression models for bacteriophage data. Univariate analysis of correlations between bacteria and bacteriophage was performed using Kendall’s rank correlation coefficient [51]. The correlation between bacteriophage and bacterial alpha diversity (Shannon) was determined using Pearson product-moment correlation coefficient. All analyses were performed in R and Prism version 8.3.0 for Windows (GraphPad Software, La Jolla, California USA) [52]. *p* values in the univariate analyses were adjusted for multiplicity using the Benjamini–Hochberg procedure [53]. The significance threshold for all analyses was set at *p <* 0.05 unless otherwise stated.

## 3. Results

### 3.1. Cohort Characteristics

50 HIV-positive and 50 HIV-negative young, sexually active South African women ages 16–21 were recruited in Cape Town, South Africa between October 2012 and October 2014 as part of the University of Cape Town HPV-HIV study to investigate high-risk HPV persistence in HIV-positive women as previously reported [27,38]. Vaginal swabs were self-collected at six-month intervals for up to six consecutive visits, along with medical and sexual history, laboratory data, and demographic information. Pap smears and STI testing were performed at the baseline visit and each year. HIV-positive subjects had an average CD4+ T cell count of 477.5 cells/μL, and 22 (44%) were not on highly active antiretroviral therapy (HAART) at the baseline study visit. There was an increased risk of HPV for HIV-positive participants at baseline (OR 5.299, 95% CI 2.048 to 13.73). This cohort included 54 BV-positive and 46 BV-negative subjects at baseline (Table 1). The median age of both BV-positive and BV-negative women was 19. BV-positive subjects trended toward higher rates of HPV infection with any subtype (*p* = 0.0940) and had a significantly higher prevalence of high-risk HPV subtypes (*p* = 0.0005) compared to BV-negative subjects. These data demonstrate that our cohort behaves similarly to other published cohorts and was suitable for further study [54].

### 3.2. The FRT Bacteriome Clusters into Community Groups

**Figure 1:**
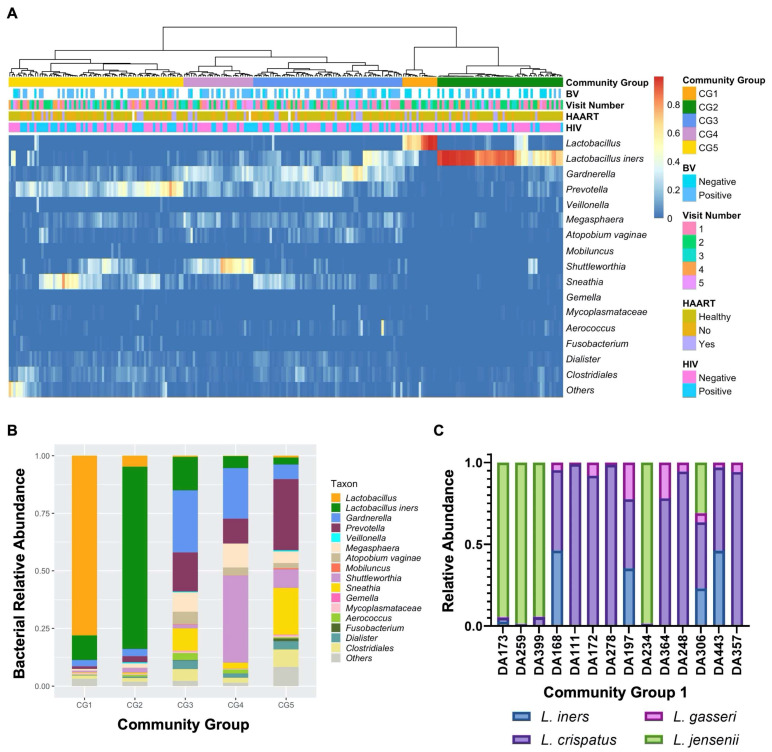
Bacteriome profiling by community group of self-collected vaginal swabs from South African women. (**A**) Relative abundance of the 16 most frequently identified bacterial taxa (y-axis) by sample (x-axis), grouped by community group (CG), bacterial vaginosis (BV) Status, visit number, highly active antiretroviral therapy (HAART) status, and human immunodeficiency virus (HIV) status (color key shown). Percent abundance is indicated by gradient key. Using Ward’s linkage hierarchical clustering, samples clustered into five distinct bacterial community profiles termed CG. (**B**) Bacterial composition (color key shown) for each of the five CGs (x-axis) expressed as relative abundance (y-axis). (**C**) Bar plot showing the relative abundance of 16S rRNA copies per 10 ng total DNA (y-axis) of *L. iners* (blue), *L. crispatus* (purple), *L. gasseri* (pink), and *L. jensenii* (green) bacterial species as determined by qPCR of FRT samples (x-axis) that clustered into CG1.

Prior studies examining Western cohorts have identified five bacterial community state types (CSTs), of which type I–III and V are *Lactobacillus*-dominant while CST IV is comprised of polymicrobial communities [8]. However, studies examining the FRT bacteriome in African cohorts have revealed a different pattern in hierarchical clustering analysis, with more community groupings of high diversity bacteriomes, consistent with the increased prevalence of high diversity FRT bacteriomes in this population [4,8,55]. To further assess the bacterial communities within the FRT bacteriome seen in African women, 253 vaginal swabs were processed and underwent 16S rRNA gene amplicon sequencing of the V3–V4 region [28]. A total of 11 samples did not amplify, and 4 failed to achieve sufficient reads for downstream analysis, leaving 238 samples for bacteriome analysis and sequence identification using QIIME2 (Figure 1A).

The 16S samples were analyzed using VALENCIA [36], a program developed to assign 16S vaginal samples to the commonly used CST communities [8] and employs similarity scores ranging from 0 (no shared taxa) to 1 (all taxa shared and at the same relative abundance) to assess assignment confidence. VALENCIA successfully assigned *L. iners*-dominant samples to CST III (*L. iners*-dominant CST) with high confidence (Supplementary Table S1; similarity score 91%). However, the remaining CST assignments were low confidence with an overall similarity score average of 29.7%. This likely reflects the bias of VALENCIA toward *Lactobacillus*-dominant samples. Because samples within our cohort were predominantly high diversity, we used Ward’s linkage hierarchal clustering to classify samples into distinct bacterial communities by composition and relative abundance.

**Figure 2:**
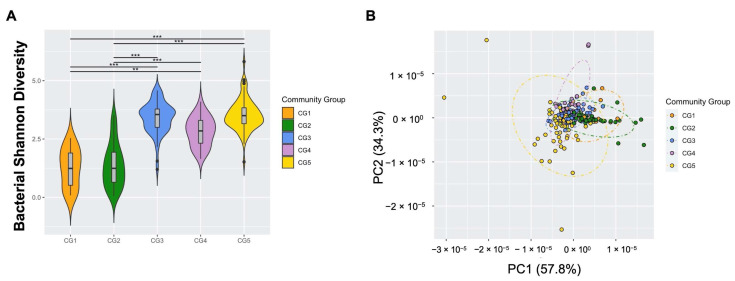
FRT bacteriome clusters into distinct community groups (CGs) that differ by alpha and beta diversity. (**A**) Bacterial Shannon diversity (y-axis) by CG (x-axis) as determined by linear mixed-effects model. Center bar represents median; gray box bounded by upper/lower interquartile ranges (IQR); whiskers represent range; dots represent outliers; color-filled areas are representative of density/distribution of diversity values. **, *p* < 0.01; ***, *p* < 0.001. (**B**) Principal coordinate analysis (PCoA) plot of the weighted UniFrac distances colored by CG (color key shown).

Hierarchical clustering analysis of all visits by community abundance and composition identified five unique bacterial community clusters named herein bacterial community groups (CGs), which were distinguished by *Lactobacillus*-dominance (CG1 and 2) or higher diversity bacteriomes (CG3–5; Figure 1A). Similar to other African cohorts [4,8,55], the majority (*n* = 173, 72.7%) of subjects had high diversity FRT bacteriomes with a low prevalence of *Lactobacillus*-dominant bacteriomes. Samples that were dominated by a single species, defined as >50% community composition, made up 42.0% of all samples and were mostly clustered with CG1 and 2, while the remaining samples showed no individual dominant species and mainly clustered in CG3–5. CG1 (*n* = 15, 6.3%; Figure 1B), a low diversity FRT bacteriome, was comprised almost exclusively of *Lactobacillus* species (78%) that were unable to be further delineated by 16S rRNA gene amplicon sequencing. To further define the predominant *Lactobacillus* constituents of CG1, qPCR of 16S rRNA gene sequences from the key FRT *Lactobacillus* species *L. iners*, *L. crispatus*, *L. gasseri,* and *L. jensenii* [9,55] was performed, revealing that approximately 50% of samples in CG1 were *L. crispatus*-dominant, similar to what has been previously described as CST I [8] (Figure 1C). One vaginal swab in CG1 contained sufficient volume to only perform qPCR for *L. iners* and *L. crispatus*, and *L. crispatus* was most abundant (not shown). *L. jensenii* tended to predominate when present (Figure 1C). CG2 (*n* = 54, 22.7%) was *L. iners*-dominant, with a few samples showing notable amounts of *Gardnerella* and *Prevotella* as well (Figure 1B). The identified high diversity CGs 3–5 were compositionally different from the conventional CST4 subtypes (Supplementary Table S1) [8]. The second to largest and most diverse group, CG3 (*n* = 64, 26.9%), consisted mainly of *Gardnerella*, *Prevotella,* and *L. iners* (Figure 1B). The smallest high diversity CG was CG4 (*n* = 30, 12.6%), in which *Shuttleworthia* and *Gardnerella* were predominant. CG5 contained the largest number of samples (*n* = 75, 31.5%) and was dominated by *Sneathia* and *Prevotella* (Figure 1B). CG3, CG4 and CG5 exhibited significantly higher alpha diversity than CG1 and 2 (Figure 2A; *p* = 0.0001, 0.0065, and <0.0001, respectively). Beta diversity significantly differed between these five bacterial CGs (Figure 2B; *p* = 0.0001).

### 3.3. Bacteriophages Comprise the Majority of the FRT DNA Virome

While the FRT bacteriome has been well-studied, the FRT virome, especially bacteriophage populations, is relatively unknown. We, therefore, characterized the FRT DNA virome using a subset of baseline samples by enriching for VLPs from resuspended vaginal swabs and extracting viral nucleic acid [47]. Libraries were constructed and sequenced using the Illumina NovaSeq platform for a subset of 38 baseline samples, 14 of which were BV-negative and 24 were BV-positive. The resulting viral sequences underwent quality control, removal of bacterial and human reads, and then were assigned to known viral taxa using VirusSeeker, a BLAST-based NGS virome analysis pipeline [40]. On average, there were 29 million reads per sample, 86.8% of them being high quality, with approximately 58% unique reads per sample.

The DNA eukaryotic virome was comprised almost entirely of *Papillomaviridae* (94% of eukaryotic viral reads). *Herpesviridae* (including herpes simplex virus, cytomegalovirus, and Epstein Barr virus) comprised 5% of viral reads, while *Polyomaviridae*, *Poxiviridae* (mulluscum contagiosum virus), and *Anelloviridae* combined were less than 1%. There was no significant difference in reads assigned to DNA eukaryotic viruses by BV or HIV status after correction for multiple comparisons. However, FRT bacteriophages were abundant, representing 83% of virally assigned reads. Samples contained sequences identified as belonging to the *Myoviridae, Siphoviridae, Podoviridae, Inoviridae, Ackermannviridae, Microviridae, Lipothrixviridae, Plasmaviridae,* and *Tectiviridae* bacteriophage families. Sequences assigned to members of the *Caudovirales* order, lytic-tailed dsDNA bacteriophages, including *Myoviridae, Siphoviridae,* and *Podoviridae*, were the most abundant in all samples regardless of BV, HAART, or HIV status.

**Figure 3:**
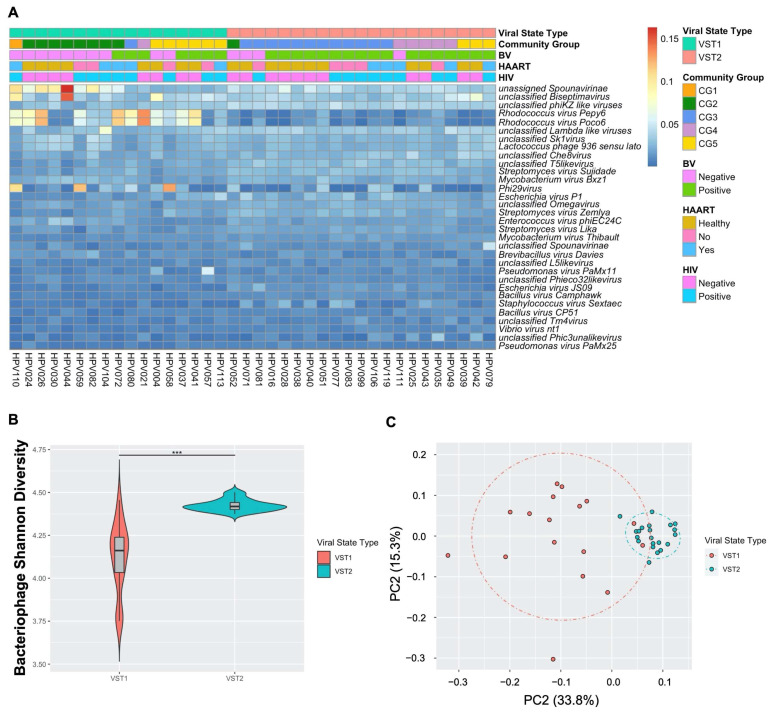
FRT DNA bacteriophages cluster into two unique community groups. Self-collected vaginal swabs were processed for DNA virome analysis by enriching for viral nucleic acid, libraries built and sequenced. (**A**) Relative abundance of the 32 most frequent bacteriophage species (y-axis) by sample (x-axis). Ward’s linkage hierarchical clustering analysis was used to cluster samples into distinct bacteriophage community profiles called viral state types (VSTs). VST, bacterial vaginosis (BV) status, highly active antiretroviral therapy (HAART) status, and human immunodeficiency virus (HIV) status (color key) are shown. Percent abundance is indicated by gradient key. (**B**) Bacteriophage Shannon diversity (y-axis) by VST (x-axis) as determined by linear regression model. Center bar represents median; gray box is bounded by upper/lower interquartile ranges (IQR); whiskers represent range; dots represent outliers; color-filled areas are representative of density/distribution of diversity values. ***, *p* < 0.001. (**C**) Principal coordinate analysis (PCoA) plots of beta diversity distances, as determined by permutational multivariate analysis of variance, colored by VST.

Bacteriophages within the FRT are clustered into two distinct, novel bacteriophage community groups based on composition and abundance that we have termed viral state types (VSTs; Figure 3A). VST1 represented 44.7% (*n* = 17) of all samples while VST2 contained the remaining 55.3% (*n* = 21). Bacteriophage Shannon diversity differed between VSTs (Figure 3B), with VST2 having the highest diversity bacteriophage populations. The VSTs were also grouped distinctly by beta diversity analysis (Figure 3C; PERMANOVA *p* = 0.0001). Neither VST exhibited a dominant bacteriophage member. VST1 contained several samples with a high relative abundance of *Rhodococcus viruses*, *Spounavirinae*, *phi29 virus* and *Biseptimavirus*-assigned bacteriophage reads. VST2 composition was more evenly distributed. This is the first study to identify bacteriophage community groups in the FRT.

### 3.4. Transkingdom Associations within the FRT Microbiome of South African Women

Bacteriophages can directly impact bacterial composition and abundance through infection of their host. Therefore, we investigated the transkingdom associations between bacteriophage and bacteria in the FRT. The VSTs significantly correlated with bacterial CG (*p* = 0.00015; Figure 3A), with VST1 associating with the *Lactobacillus*-dominant CG1 and 2, while VST2 associated with CG3 and 4, both higher diversity CG. CG5 contained samples belonging to both VSTs (Figure 3A). These data indicate a strong association between bacteriophage communities and the host bacterial populations.

**Figure 4:**
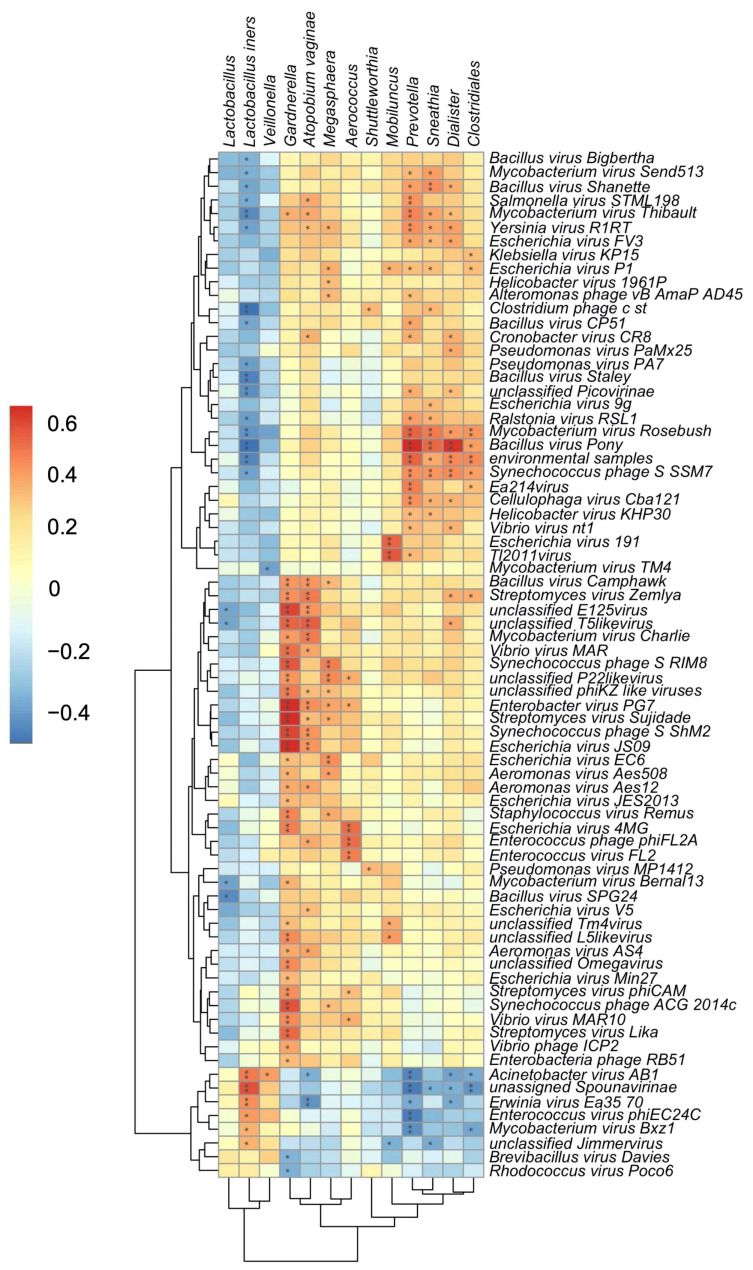
Transkingdom associations within the FRT of South African women. Heatmap of estimated Kendall’s correlation coefficients between FRT bacterial taxa (x-axis) and sequences assigned to bacteriophage (y-axis). Asterix indicate significant correlations after multiple comparisons correction by the Benjamini–Hochberg procedure, *, *p* < 0.05; **, *p* < 0.01. Magnitude and sign of the Kendall’s rank correlation coefficient are indicated by gradient key. Red indicates positive correlations; blue indicates negative correlations.

The transkingdom interplay between bacteriophage and bacteria in the FRT was further supported by a significant correlation identified between bacteriophage and bacterial alpha diversity (Supplementary Figure S1; *p* = 0.00032). To further investigate specific bacteriophage-bacterial interactions, correlations between FRT bacterial and bacteriophage composition were identified by Kendell’s rank correlation coefficient. Reads assigned to bacteriophages *Bacillus virus Camphawk* and *Bacillus virus Pony,* which infect members of the *Bacillus* genus, positively correlated with the BV-associated bacteria *Gardnerella, A. vaginae*, *Prevotella*, *Sneathia,* and *Dialister* (Figure 4). Reads assigned to unclassified bacteriophages of the *E125* genus are also positively associated with *Gardnerella* and *A. vaginae* (Figure 4). A number of *Bacillus*-infecting bacteriophages, including *Bacillus virus Pony and Bacillus virus Staley,* were inversely associated with bacteria protective from BV, particularly *L. iners* (Figure 4). Interestingly, in this cohort, bacteriophage associations revealed that *Veillonella* more closely grouped with *Lactobacillus* rather than BV-associated bacteria despite the known role of *Veillonella* in lactose fermentation and higher diversity FRT microbiomes [8]. These data suggest that bacteriophages directly or indirectly interact with FRT bacterial populations in disease states and identify putative FRT bacteriophage-host networks that may play a role in the development and maintenance of BV.

### 3.5. Effects of Bacterial Vaginosis on the FRT Virome

**Figure 5:**
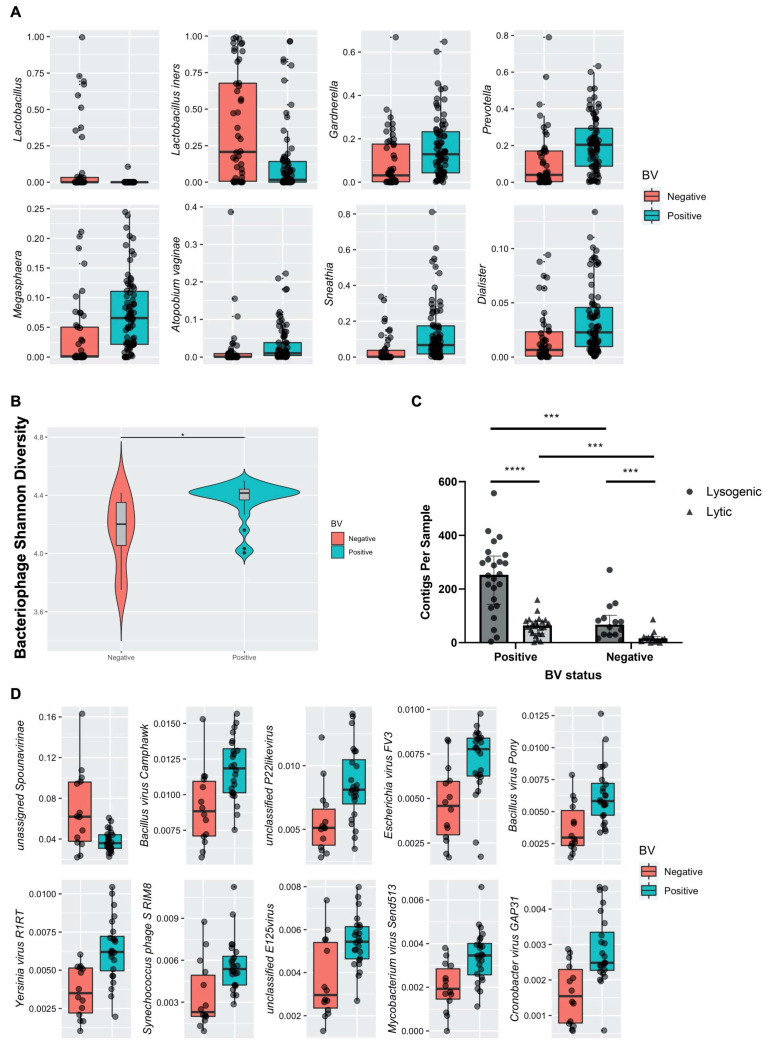
Discriminant FRT bacterial and bacteriophage species associated with bacterial vaginosis. (**A**) Significantly discriminant bacterial taxa by BV status were determined by univariate analysis using mixed-effects models. Relative abundance of each taxon (y-axis) is shown in BV-negative (orange) and BV-positive subjects (green; x-axis). All bacterial taxa presented are significant with an FDR-adjusted *p* < 0.05. (**B**) Bacteriophage Shannon diversity (y-axis) by BV status (x-axis) as determined by a linear regression model. *, *p <* 0.05. (**C**) Grouped bar plot showing the number of identified lytic and lysogenic bacteriophage contigs per sample by BV status. Lytic genes are represented as black triangles and lysogenic genes as black circles. Significance assessed using Mann–Whitney test with multiple comparisons correction by the Benjamini–Hochberg procedure. ***, *p <* 0.001; ****, *p <* 0.0001. (**D**) Significantly discriminant bacteriophage species by clinical BV diagnosis was determined by univariate analysis using a linear regression model. All bacteriophage taxa presented are significant with an FDR-adjusted *p* < 0.05.

We next examined the impact of different disease states on this cohort. BV is a clinically significant condition with high morbidity characterized by high bacterial diversity [13,14]. Our data showed significant associations between high diversity bacterial CGs and VSTs, and further suggested specific bacteriophage interactions with BV-associated bacteria. Similar to published cohorts [2], BV in our cohort was positively associated with increased bacterial alpha diversity compared to healthy subjects (*p* = 0.0001). The high diversity CG3–5 (*p* = 0.0001) also correlated positively with BV. Bacterial taxa, including *Gardnerella, Prevotella, Sneathia,* and *Megasphaera* (Figure 5A, Supplementary Table S2A), varied significantly by clinical BV status, corroborating distinct bacterial signatures in BV. We next sought to identify specific bacterial taxa associated with recovery and transition to BV that could be predictive of the dynamic changes in bacteriome profiles between health and BV. We observed a significant increase in relative abundance of the Gram-positive, facultatively anaerobic cocci *Aerococcus* (*p* = 0.00216) and *Gemella* (*p* = 0.00746) among participants who recovered from or transitioned to BV, suggesting these may be useful clinical biomarkers of impending change in BV status. While these bacteria are less frequently detected in BV [56], they may represent regulators of FRT bacteriome structure in health and BV.

Since clinical BV was associated with significant changes in FRT bacterial composition and correlated with bacterial CGs, and bacteriophage VSTs also correlated with bacterial CGs, we sought to identify correlations between bacteriophage populations and clinical BV. Regression analysis accounting for HIV status and VST revealed bacteriophage Shannon diversity significantly differed by clinical BV diagnosis (Figure 5B; *p* = 0.0193). To further investigate the manner in which bacteriophage could be affecting bacterial populations in BV, we assessed assembled contigs for the presence of lytic or lysogenic bacteriophage genes to determine bacteriophage lifestyle (Figure 5C). BV-positive samples had significantly more contigs per sample containing lytic (*p* = 0.000283) and lysogenic (*p* = 0.000283) genes compared to BV-negative samples (Figure 5C). Moreover, in both BV-positive and BV-negative participants, there were considerably more contigs containing genes associated with a lysogenic lifecycle than those identified as lytic (Figure 5C). We then ascertained specific bacteriophage taxa differentially abundant in the FRT of BV-positive and -negative women. *Bacillus*-infecting bacteriophages are known to belong to the *Herelleviridae* and *Podoviridae* families [57,58]. Members of these families, including *Bacillus virus Camphawk* and *Bacillus virus Pony* (Figure 5D, Supplementary Table S2B), were significantly associated with BV diagnosis (*p*= 0.019058 and *p =* 0.014546, respectively). BV diagnosis also strongly correlated with the bacteriophages *Escherichia virus FV3* and *unclassified E125 virus* (Figure 5D, Supplementary Table S2B), which are known to infect the BV-associated bacteria *Escherichia coli* and *Burkholderia*, respectively [59,60]. Together, these data uncover a link between highly diverse FRT bacteriophage populations, a distinct subset of bacterial hosts, and BV.

### 3.6. The Effect of HIV and HPV on the FRT Microbiome

We also examined the effect of HIV on the FRT microbiome in this cohort. We found no significant difference in bacterial richness, alpha or beta diversity between HIV-positive and HIV-negative subjects. Further, there were no significant alterations in bacteriome or bacteriophage diversity by HIV status. Thus, FRT bacterial and bacteriophage communities were not detectably altered by HIV infection in this cohort, suggesting that localized infections may play a more important role in the FRT composition than systemic infections. We also examined the relationship between HPV, the main eukaryotic virus found, and bacterial populations. Upon examination of 63 HPV-positive and 37 HPV-negative baseline samples, there were no significant associations between HPV infection and bacteriophage or bacterial diversity. Analysis of HPV subtypes revealed increased bacterial alpha diversity in HPV6-positive subjects (*p* = 0.0181), suggesting certain HPV subtypes may directly or indirectly benefit from the presence of higher diversity bacterial populations, although this analysis may have been underpowered for less prevalent subtypes.

## 4. Discussion

The FRT is a dynamic ecosystem in which bacteriophage and bacterial communities establish complex connections that influence the host environment and the physical manifestation of gynecological diseases. Changes in bacteriome composition are well-established contributors to FRT disease states, including BV, pre-term birth, and STI acquisition [1,6,8]. However, little is known about the FRT viral populations. This study offers a comprehensive characterization of both the FRT bacteriome and DNA virome, with a particular emphasis on bacteriophage composition and transkingdom interplay, using a South African cohort of BV-affected women. This is the first study to describe novel bacteriophage communities we have termed VSTs that significantly associate with bacterial communities and clinical BV diagnosis. Although bacteriophages have been studied at other mucosal sites in humans, including the gut [61], this is the first description of bacteriophage community groupings, which may be unique to the FRT environment due to the distinctive bacterial communities present.

Few prior studies have examined the bacteriophage populations in the FRT, but when bacteriophages have been evaluated, no distinct viral communities were found nor significant difference in viral or bacterial alpha diversity between BV-positive and BV-negative samples in these studies [4,25]. However, the significantly increased sequencing depth used in our study, analysis of all DNA bacteriophages, combined with updated virus databases, likely explains differences observed between these studies and ours. Our viral sequencing depth of 29 million reads per sample was greater than 640-fold higher than that employed in Jakobsen et al. (average of 44,686 reads per sample) [25] and is likely a major contributor to our unique findings. Validation of our novel FRT VSTs using other cohorts is currently underway.

Our analysis also identified discriminant bacteriophage taxa by BV status and assessed transkingdom associations in the FRT. Correlation analysis revealed bacteriophages that positively correlated with BV-associated bacteria and inversely correlated with *Lactobacillus*, suggesting that transkingdom interactions between bacteriophages and bacterial species could be the driver of BV-associated bacterial community alterations. *Bacillus virus Camphawk* and *Bacillus virus Pony*, previously demonstrated to be lytic to *Bacillus* members [62,63], were associated with both clinical BV diagnosis and BV-associated bacteria. In the gut, *Bacillus* strains have been shown to antagonize enteropathogenic bacteria while concurrently promoting the growth of *Lactobacillus* [64]. If *Bacillus* species act in a similar manner in the FRT, the lytic nature of the *Bacillus* bacteriophages *Bacillus virus Camphawk* and *Bacillus virus Pony* could at least partially explain the shift in vaginal microbiota away from *Lactobacillus* species and toward more diverse bacterial species, including the facultative anaerobes seen in BV. In addition, of note, we found that *E. coli* and *Burkholderia* bacteriophages *Escherichia virus FV3* and *unclassified E125* virus, respectively, were associated with clinical BV diagnosis. While less predominant than other bacterial species, *E. coli* and *Burkholderia* have also been implicated in BV-associated bacterial communities [2,18,59]. *Escherichia virus FV3* and *unclassified E125* virus may act to regulate the bacterial abundance and community composition in BV via a predator-prey relationship to allow for growth of primary BV-associated bacterial members such as *Gardnerella* and *Prevotella* [24,65]. Additional investigation of other transkingdom contributors in the FRT environment, such as fungi, could further illuminate mechanisms of BV pathogenesis.

We further examined FRT bacteriophage contigs to identify bacteriophage lifestyle and found the majority of identified contigs contained genes consistent with lysogeny. A recent study using metagenomic sequencing to examine the FRT microbiome in South African adolescents also found a high proportion of identified lysogenic phages in the FRT [26]. However, the larger sample size in our study allowed us to further evaluate for the difference by clinical BV diagnosis, revealing significantly more contigs containing lysogenic genes (including integrase, transposase, repressor, and recombinase genes) in BV-positive than BV-negative samples. These data suggest that lysogenic bacteriophages could play a significant role in shaping bacterial communities, particularly in BV. Since lysogenic bacteriophages can carry genes capable of enhancing the virulence of their bacterial hosts, they may provide survival advantages through mechanisms such as conferring tolerance to ecological stressors, immunity from superinfection, increased pathogenicity, or antibiotic resistance [66,67]. The high proportion of lysogeny we found in BV could reflect the contribution of these bacteriophages to BV-associated bacterial stability, antimicrobial resistance to BV treatment, or the high BV recurrence rates of up to 50% despite successful initial treatment [68,69,70,71]. BV risk factors such as new or multiple sexual partners provide a plausible mechanism for the introduction of novel lytic and lysogenic bacteriophages that could target and modulate FRT bacterial community structure and composition [72,73].

Interestingly, no discriminant bacteriophages ascribed to the BV-associated bacterial hosts *Gardnerella*, *Prevotella,* or health-associated *Lactobacillus* were found. The absence of bacteriophages that infect hallmark BV bacteria such as *G. vaginalis* in our analysis may be attributable to the number of CRISPR/Cas defense loci found in *Gardnerella* species, which could provide more resistance to bacteriophage infection and establish an uneven bacteriophage burden between bacteria in BV [74]. A similar trend has also been seen among a number of FRT *Lactobacillus* species, including *L. iners*, *L. crispatus,* and *L. jensenii,* which have been shown to contain CRISPR spacers and upregulation of CRISPR-related genes in BV, and could explain why *Lactobacillus* bacteriophages were not observed in our analysis [75,76]. Other possible explanations include methodological biases. The initial steps of our viral nucleic acid enrichment method are designed to remove contaminating bacteria and bacterial DNA. Since prior studies have found that *Lactobacillus* species harbor a high proportion of lysogenic bacteriophages [77,78,79,80], removal of bacterial DNA may have also removed prophage sequences, thus explaining the few *Lactobacillus* bacteriophages found. This seems an unlikely explanation since, of identifiable contigs in our study, lysogeny prevailed. Another possibility is that since fewer *Lactobacillus*-dominant samples were sequenced, and fewer bacteriophage-identified contigs were found in BV-negative samples, greater sequencing depth or sequencing of more *Lactobacillus*-dominant samples may be required to characterize FRT *Lactobacillus* bacteriophages. Further metagenomic sequencing and in vitro studies will be necessary to establish the host range and the contribution of CRISPR defense loci to bacteriophage dynamics in the FRT.

Limitations to this study include the cross-sectional nature of the virome analysis preventing speculation on the longitudinal impact of bacteriophage changes and the inability to assess the FRT RNA virome due to low RNA integrity of the samples. Additionally, 16S rRNA sequencing was unable to resolve bacterial taxonomy to species level in some cases, a common deficiency of this method. The initial limited patient consent also precluded significant in vitro validation of bacteriophage-bacterial pairs or analysis of localized inflammation. Finally, there may be inaccuracies or biases in sequence assignments.

## 5. Conclusions

In this retrospective longitudinal study, we performed a novel in-depth characterization of the FRT virome and bacteriome using a cohort of young, sexually active, South African women. We discovered significant alterations in FRT bacterial and bacteriophage diversity and community structure associated with BV. Transkingdom analysis revealed associations of specific bacteriophages with bacteria protective of and associated with clinical BV diagnosis. This study is the first to describe the VST bacteriophage community structure within the FRT and its associations with bacterial diversity and composition. The nature of the FRT microbiome in health and disease is both complex and dynamic, and our findings provide insight into putative interactions between bacteriophage and bacteria that may contribute to the development and maintenance of FRT dysbiosis. Further studies are needed to investigate direct mechanisms employed by bacteriophages to promote dysbiosis.

## Figures and Tables

**Table 1:** Cohort characteristics.

Cohort Characteristics	BV-Positive (*n* = 54)	BV-Negative (*n* = 46)	*p*-Value
Age (Years), Mean (Interquartile Range; IQR)	19.2 (16–21)	18.8 (16–21)	0.2352
**Laboratory Results**			
HIV-Positive Samples, *n* (%)	29 (53.70)	21 (45.65)	0.5475
HPV-Positive Samples, *n* (%)	39 (60.94)	25 (39.06)	0.0940
High-Risk HPV Subtypes Present in Positive Samples, *n* (%)	29 (76.32)	9 (23.68)	0.0005
Visits with Abnormal Pap Smear, *n* (%)	13	5	0.1181
**Smoking History**			
Smoker, *n* (%)	5 (5)	4 (4)	>0.9999
Non-Smoker, *n* (%)	49 (49)	42 (42)	
**Sexual History**			
History of STI, *n* (%)	27 (57.45)	20 (42.5)	0.5514
Lifetime Sexual Partners			
1, *n* (%)	11 (11)	4 (4)	0.2482
2–5, *n* (%)	39 (39)	39 (39)	
>5, *n* (%)	4 (4)	3 (3)	
Sexual Partners in the Last 6 Months			
1, *n* (%)	51 (51)	44 (44)	>0.9999
2–5, *n* (%)	3 (3)	2 (2)	
Form of Contraception			
None, *n* (%)	1 (1)	1 (1)	0.3712
Condom, *n* (%)	50 (50)	40 (50)	
Injection, *n* (%)	30 (30)	31 (31)	
Pill, *n* (%)	2 (2)	3 (2)	

HIV, human immunodeficiency virus; HPV, human papilloma virus; STI, sexually transmitted infection. High-risk HPV subtypes are defined as those that are carcinogenic, including subtypes 16, 18, 31, 33, 35, 39, 45, 51, 52, 56, 58, 59, 68, 73, and 82. For continuous variables, Mann–Whitney and Kruskal–Wallis tests were used; for comparing categorical variables, chi-square and Fisher’s exact tests were used.

## Data Availability

The microbiologic sequencing data presented in this study are openly available in the SRA database (ascension number: PRJNA726546).

## Supplementary Materials

The following supporting information can be downloaded at: https://www.mdpi.com/article/10.3390/v14020430/s1, Figure S1: Transkingdom Correlation between Bacterial and Bacteriophage Diversity in the FRT; Table S1: Comparison of Community Group (CG) and Community State Types (CST); Table S2A: Statistical values for Figure 5A taxa; Table S2B: Statistical values for Figure 5D taxa.

## Abbreviations

BV	Bacterial Vaginosis
CG	Bacterial Community Group
DNA	Deoxyribonucleic Acid
HAART	Highly Active Antiretroviral Therapy
HIV	Human Immunodeficiency Virus
HPV	Human Papilloma Virus
FRT	Female Reproductive Tract
NGS	Next-Generation Sequencing
PERMANOVA	Permutational Multivariate Analysis of Variance
STI	Sexually Transmitted Infection
VLP	Virus-Like Particle
VST	Viral State Type
