# Supplementary material for: Transkingdom Analysis of the Female Reproductive Tract Reveals Bacteriophages form Communities
Source: Viruses. 2022 Feb 19;14(2):430. doi: 10.3390/v14020430 (PMC8878565; doi:10.3390/v14020430)
Supplement: Supplementary file 1 [file viruses-14-00430-s001.zip › viruses-1599144-supplementary.pdf]

Supplement to:

Transkingdom Analysis of the Female Reproductive Tract Reveals Bacteriophages Form Communities

Ferralita S. Madere[1], Michael Sohn[2], Angelina Winbush[3], Breóna Barr[4], Alex Grier[5], Cal Palumbo[5], James Java[5], Tracy Meiring[6], Anna-Lise Williamson[6,7], Linda-Gail Bekker[8], David H. Adler[9], Cynthia L. Monaco[1,10*]

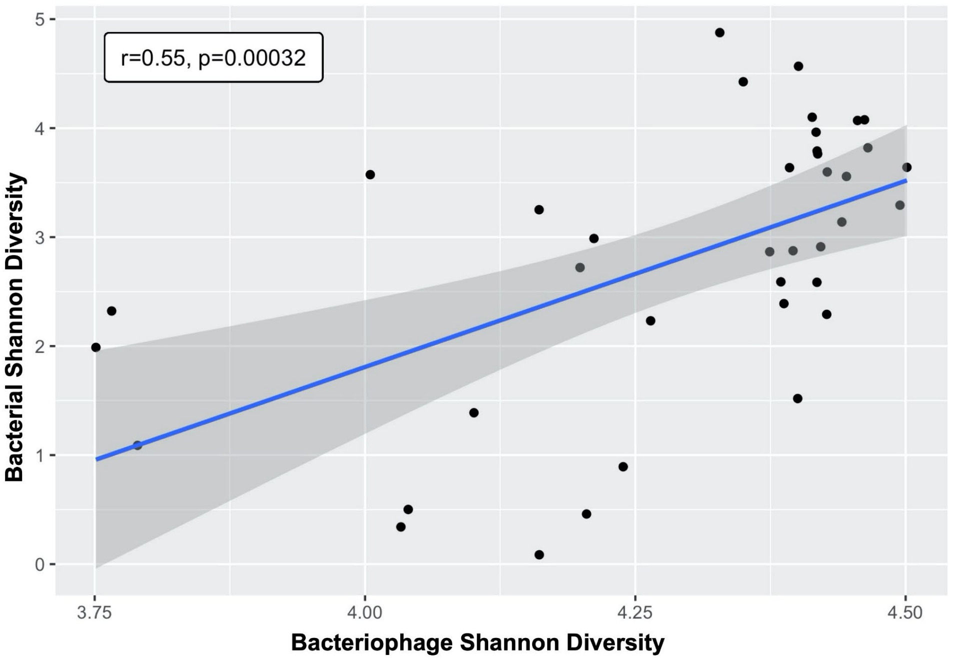

Supplementary Figure S1: Transkingdom Correlation between Bacterial and Bacteriophage Diversity in the FRT. Scatter plot showing the correlation with 95% confidence interval (dark grey) of the regression line (blue) between the sample bacterial and bacteriophage Shannon diversity.

Supplementary Table S1: Comparison of Community Group (CG) and Community State Types (CST)

	CG 1	CG 2	CG 3	CG 4	CG 5
**Total N**	15	54	64	30	75
**CST III**	8	54	13	1	7
**CST IV-A**	0	0	0	1	27
**CST IV-B**	5	0	51	28	36
**CST IV-C**	2	0	0	0	5
**Average Similarity Score**	0.0882	0.9180	0.5218	0.3174	0.1330

Average VALENCIA similarity score (not including CG2): 0.2966

Supplementary Table S2A: Statistical values for Figure 5A taxa

	p-val (BV)	FDR adj. *p*-val (BV)
Lactobacillus	0.000112	0.000475
Lactobacillus iners	0.000281	0.000955
Gardnerella	0.005973	0.014506
Prevotella	7.70E-05	0.000439
Megasphaera	4.10E-05	0.000439
Atopobium vaginae	0.012197	0.025918
Sneathia	7.30E-05	0.000439
Dialister	0.000362	0.001024

Supplementary Table S2B: Statistical values for Figure 5D taxa

	p-val (BV)	FDR adj. *p*-val (BV)
**unassigned_*Spounavirinae***	0.001167	0.019058
Bacillus_virus_Camphawk	0.001137	0.019058
**unclassified_P22likevirus**	0.002274	0.028879
Escherichia_virus_FV3	0.001896	0.027874
Bacillus_virus_Pony	0.000364	0.014546
Yersinia_virus_R1RT	0.000164	0.014546
Synechococcus_phage_S_RIM8	0.00106	0.019058
**unclassified_E125virus**	0.000535	0.015739
Mycobacterium_virus_Send513	0.001025	0.019058
Cronobacter_virus_GAP31	0.000216	0.014546
